# Association between change in lifestyle and cognitive functions among elderly Koreans: findings from the Korean longitudinal study of aging (2006–2016)

**DOI:** 10.1186/s12877-020-01693-7

**Published:** 2020-08-31

**Authors:** Hyeon Ji Lee, Jieun Jang, Dong-Woo Choi, Wonjeong Chae, Eun-Cheol Park, Sung-In Jang

**Affiliations:** 1 Department of Public Health, Graduate School, Yonsei University, Seoul, Republic of Korea; 2 Institute of Health Services Research, Yonsei University, 50 Yonsei-ro, Seodaemun-gu, Seoul, 03722 Republic of Korea; 3 Department of Preventive Medicine and Public Health, Ajou University College of Medicine, Suwon, Republic of Korea; 4 Department of Preventive Medicine, Yonsei University College of Medicine, Seoul, Republic of Korea

**Keywords:** Aging, Cognitive function, Elderly people, Health behavior, Lifestyle

## Abstract

**Background:**

South Korea is an aged society that continues to age rapidly. Therefore, the purpose of this study was to investigate the association between changes in lifestyle and cognitive functions in the South Korean elderly using a nationally representative survey.

**Methods:**

We analyzed data from the Korean Longitudinal Study of Aging (KLoSA) 2006–2016, a biannual panel survey. Multiple linear regression analysis was performed with repeated measurements data to examine the association between lifestyle change and cognitive functions over 2 years. Lifestyle combined the scores of four factors (smoking status, alcohol drinking status, body weight, and exercise), and then categorized them into four groups (Good→Good, Bad→Good, Good→Bad, and Bad→Bad) according to the two-year change. Cognitive functions were set according to the scores measured through the Korean Mini-Mental State Examination (K-MMSE).

**Results:**

Among females, the K-MMSE score was the highest in the Bad→Good group compared to the reference group, Bad→Bad (β = 0.914; SD = 3.744; *p* < .0001). The next highest scores were in the Good→Good group (β = 0.813; SD = 4.654; *p* = 0.0005) and the Good→Bad group (β = 0.475; SD = 4.542; *p* = 0.0481). Among males, only the K-MMSE of the Good→Good group was statistically significant (β = 0.509; SD = 3.245; *p* = 0.0077). The results of subgroup analysis showed that the K-MMSE scores of females who did not participate in any social activities were more affected by their lifestyle (Good-Good: β = 1.614; SD = 4.270; *p* = 0.0017, Bad-Good: β = 1.817; SD = 3.945; *p* < .0001). Subgroup analysis showed that females who started drinking more than a moderate amount of alcohol had lower K-MMSE scores (Good-Bad: β = − 2.636; SD = 2.915; *p* = 0.0011). Additionally, in both sexes, exercising, among the four lifestyle options, had a strong and significant association with higher K-MMSE scores.

**Conclusions:**

Following a healthy lifestyle or improving an unhealthy lifestyle can help people prevent or slow down cognitive decline. Regularly engaging in an adequate amount of exercise can help the cognitive function of the elderly. Females, specifically, can experience positive effects on their cognitive function if they participate in social activities while maintaining healthy lifestyles, in particular not drinking too much alcohol.

## Introduction

South Korea entered an “aged society” in 2018, when nearly 14.3% of its total population were over 65 years old [1]. It took 17 years to reach this status, having been designated an “aging society” in 2000. Although population aging is a problem that most countries are now facing, as life expectancy increases [2], South Korea is currently showing the fastest aging trend of any country. In light of population aging, the concept of “healthy aging” has become increasingly important in South Korea. The World Health Organization (WHO) established “healthy aging” as the ideal aging direction for 2015–2030, and defined it as the maintenance of functional abilities in elderly adults to ensure their well-being through consideration of mental capacity, physical capacity, and environment [3].

Mental health is essential for healthy aging. One to the greatest threats to mental health in elderly adults is dementia or a decrease in cognitive function. In January 2018, about 50 million people had dementia worldwide [4], and this population is estimated to swell to around 152 million by 2050 [5]. In South Korea, around 702,436 elderly people had dementia in 2017 (9.94% of the total elderly population), with an annual disease burden of about 20.74 million won (1 US dollar = 1123 Korean won) [6]. This population is expected to increase exponentially, reaching 1 million by 2024 and over 2 million by 2041. By 2040, the disease burden of dementia is estimated to be close to 78 trillion won [6].

Since cognitive decline is mainly irreversible, it is important to prevent its onset, or at least slow its pace [7, 8]. The major causes of cognitive decline leading to dementia include heredity, family history of dementia, brain trauma, disability, and lifestyle. Among these, lifestyle is relatively easy to modify, and fundamental at the same time [9]. Therefore, many researchers have explored the relationship between lifestyle changes and cognitive function. For instance, lifestyle factors such as sleep, smoking, alcohol drinking, body weight, nutrition status, and exercise all influence cognitive function or dementia [10–16]. Smoking, alcohol drinking, unhealthy body weight, and physical inactivity are also predictive factors of healthy aging among the elderly [10–12], and are highly likely to co-occur within an individual [13]. One study even combined these factors to express a “lifestyle risk score,” and examined its relationship with activities of daily living (ADLs) and instrumental ADL (IADLs) [17]. They found that lifestyle changes do influence physical disability, which is known to be related to cognitive function in the elderly [18]. One systematic review article reported the association between sedentary behavior and cognitive function [19], and suggested proper sedentary time and regular exercise to improve cognitive function. However, the study did not include South Korean data.

Therefore, our study aimed to examine the association between lifestyle changes and cognitive functions using data representing the South Korean elderly. In this study, we interpreted the changes in lifestyle risk scores [17] as changes in lifestyle. We also focused on identifying which lifestyle factors have the strongest association with cognitive function among elderly adults.

## Methods

### Data collection and participants

We obtained data for this longitudinal study from the first (2006) to sixth (2016) waves of the Korean Longitudinal Study of Aging (KLoSA). The KLoSA is a nationally representative panel survey conducted biennially through computer-assisted personal interviewing (CAPI). The survey targets community-dwelling adults aged 45 years or older living in 15 large administrative areas in South Korea. The survey target was randomly selected using a multistage and stratified sampling method by sorting survey districts stratified by region and residential type in order of administrative code. The KLoSA survey includes topics that are considered to affect economic and social activities of middle-old/aged people. The topic consists of seven categories: demographics, family, health, employment, income, assets, and subjective expectations and satisfaction. The first wave of the KLoSA (2006) included 10,254 participants, and the second wave of the survey (in 2008) included 8688 of participants from the first wave (84.7% of the primary panel). The third wave of the survey (in 2010) included 7920 (77.2%) participants, and the fourth wave of the survey (in 2012) included only 7486 of participants from the first wave (73.0%). Hence, 920 people were added to the fifth survey in 2014. A total of 7949 people (among them 7029 people were original panel) were surveyed in the fifth wave, and 7490 (6618 people were original panel) in the sixth wave (2016). In this study, we used the first to sixth wave of the original panel data for analysis. Detailed information about the survey is available on the panel survey organization website (https://survey.keis.or.kr/eng/klosa/klosa01.jsp).

**Fig. 1:**
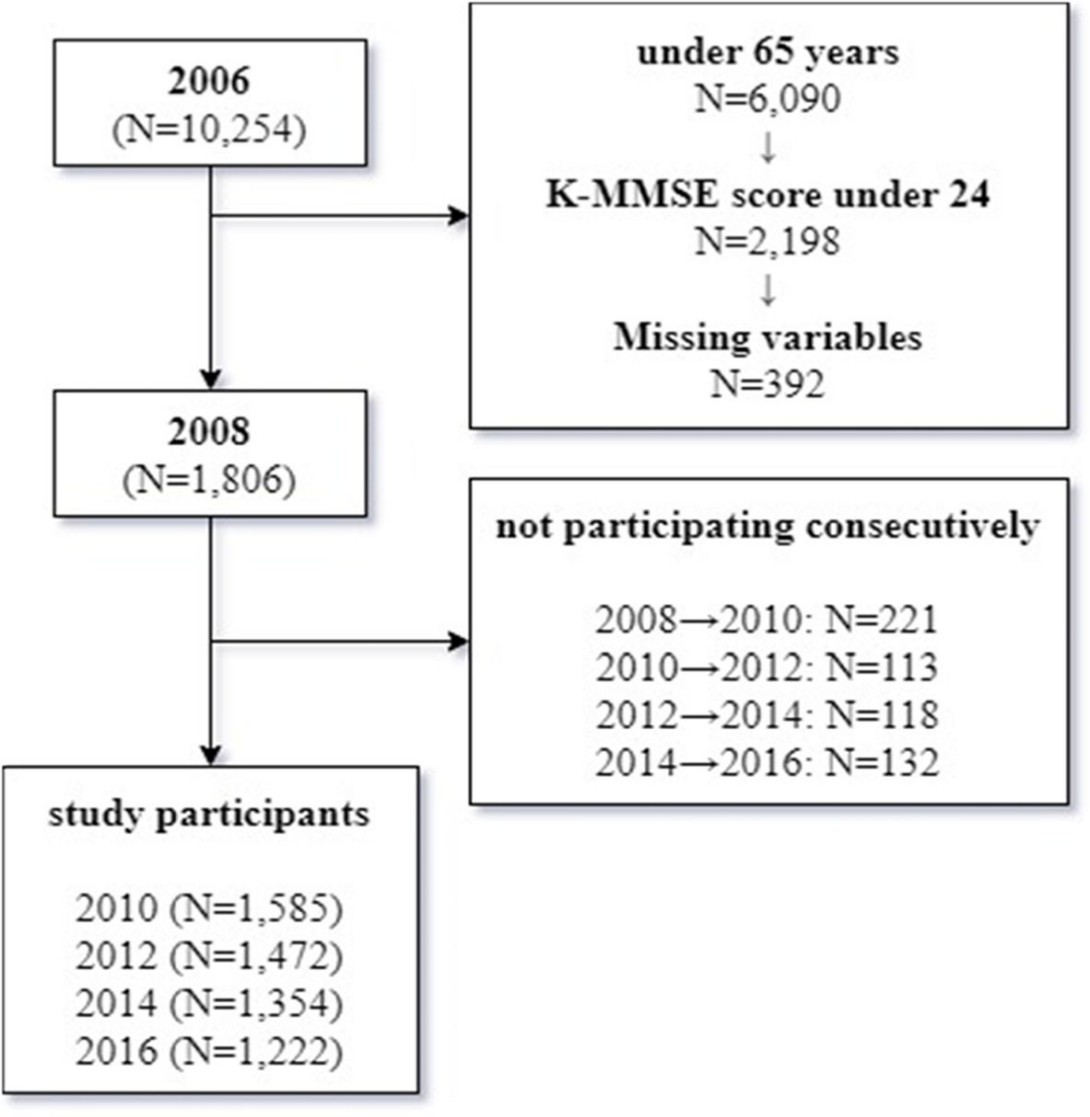
Flow chart of study participants. We excluded 6090 participants under 65 years in age from the first wave to ensure that we analyzed only individuals 65 years or older (*n* = 4164). Additionally, we excluded participants with cognitive impairment (Korean Mini-Mental State Examination [K-MMSE] score under 24) at the first wave (*n* = 2198). We also excluded participants who did not complete the questions on the independent variables of interest (e.g., socioeconomic status, social activity participation, and health-related variables) or the dependent variable (K-MMSE). To analyze changes in variables over time, we excluded participants who did not participate in more than two follow-up surveys. Ultimately, 1806 people were analyzed in 2008, 1585 in 2010, 1472 in 2012, 1354 in 2014, and 1222 in 2016

We excluded 6090 participants under 65 years in age from the first wave to ensure that we analyzed only individuals 65 years or older (*n* = 4164). Additionally, we excluded participants with cognitive impairment (Korean Mini-Mental State Examination [K-MMSE] score under 24) at the first wave (*n* = 2198). We also excluded participants who did not complete the questions on the independent variables of interest (e.g., socioeconomic status, social activity participation, and health-related variables) or the dependent variable (K-MMSE). To analyze changes in variables over time, we excluded participants who did not participate in more than two follow-up surveys. Ultimately, 1806 people were analyzed in 2008, 1585 in 2010, 1472 in 2012, 1354 in 2014, and 1222 in 2016 (Fig. 1).

### Cognitive function

The dependent variable was cognitive function as assessed via the K-MMSE [20]. The K-MMSE assesses time orientation, spatial orientation, memory registration, attention and calculation, memory recall, language, and space-time functions by trained interviewers. It has a total score of 30, with higher scores indicating better cognitive function. Scores of less than 18, 18–24, and more than 24 are defined as dementia, mild cognitive impairment, and normal, respectively [21, 22].

### Changes in lifestyle risk score

The main independent variable was changes in lifestyle risk score [17]. Lifestyle risk score was calculated from participants’ smoking status, alcohol drink status, body weight, and exercise participation. We first calculated participants into risk and risk-free groups for each factor: if they had smoked more than 100 cigarettes in their lifetime or were currently smoking [23], drank more than three drinks a day or seven drinks or more per week for any type of alcohol [24], (e.g., soju, beer, wine, raw rice wine, whiskey), had a body mass index (BMI) of < 18.6 (underweight) or ≥ 30 (overweight) [25], or engaged in less than 150 min of exercise per week [26], they were placed in the risk group. Otherwise, they were considered risk-free. A score of 1 was assigned to the risk group, and a score of 0 was assigned to the risk-free group. These scores were then summed to create the lifestyle risk score (range: 0–4). Since the mean lifestyle risk score was 1.2, we categorized the participants according to whether they had 1 point or not (0 point: good, ≥1: bad). Finally, to examine cognitive function according to lifestyle changes, we classified the participants according to their pattern of changes in lifestyle factors over two years, as follows: Good→Good, Good→Bad, Bad→Good, or Bad→Bad.

### Covariates

Socioeconomic status and health-related variables known to be associated with lower cognitive function were set as covariates. Socioeconomic status variables included age, economic activity participation, region, marital status, living arrangement, educational level, household income, and participation in social activities. Household income was grouped into quartiles for each survey year. Participation in social activities was categorized according to the number of social activities of the subjects. If the participants engaged in social activities related to religion, friendship, leisure, sport, service, or politics, they were categorized as “1” or “2+” according to the number of activities they participated in; otherwise, they were categorized as “0.” Health-related variables included the presence of chronic disease, depression, and disability status. Depression was classified as “yes” if participants scored 4 or higher on the 10-item Center for the Epidemiological Studies of Depression Short Form (CES-D10) and “no” otherwise. The disability status was measured by ADL and IADL. ADL includes 7 items that are required to live an independent life, and IADL includes 10 items related to social functions. If there is any difficulty related to an item 1 point is given for each item. Disability status was divided into nondisabled (both ADLs and IADLs had a score of 0), mild (ADL has a score of 0 and IADL had a score of 1 or more), and severe (both ADLs and IADLs had scores of 1 or more) [17]. To take into account the dependent variable (K-MMSE scores) from the previous survey, the lagged dependent variable was included as a covariate.

### Statistical analysis

Using the t-test and analysis of variance (ANOVA), we examined the differences in average K-MMSE scores according to the independent variables at the first time point. Multiple linear regression analysis using the generalized estimating equation (GEE) model was performed with repeated measurements data to examine the association between lifestyle change and cognitive function over two years [27]. Each person was measured up to five times in total (2006 → 2008, 2008 → 2010, 2010 → 2012, 2012 → 2014, 2014 → 2016). We used the SAS procedure PRCO GENMOD to analyze repeated measurements. This analysis yielded beta coefficient and their standard errors (SE), along with the *P*-value. P-value < 0.05 was considered significant. We also tested for multicollinearity in the statistic model. The variance inflation factor (VIFs) were all less than 10, indicating no excessive correlations between the independent variables in the statistical model. In addition, post-hoc analysis was performed using the Bonferroni correction method to identify multiple comparisons bias. All statistical analyses were performed using SAS statistical software package version 9.4 (SAS Institute Inc., Cary, NC, USA).

## Results

Table 1 shows the general characteristics of the study population at the first time point (2006 → 2008). In addition, the interesting and independent variables were expressed as mean values through the dependent variables K-MMSE score. Among males, the K-MMSE score was highest for the Good→Good group (*n* = 68), at 27.0 (SD = 2.6); especially, the lowest was found for the Good→Bad group (*n* = 47), at 24.2 (SD = 6.6). Among females, the Bad→Good group (*n* = 90) had the highest K-MMSE score, at 25.9 (SD = 3.5), while the Bad→Bad group (which was also the largest group, *n* = 519) had the lowest score, at 24.1 (SD = 4.4). Additionally, both males and females did not differ in their cognitive function scores according to their living areas in urban areas and rural areas (Male: Mean = 26.3; SD = 3.9. Mean = 25.6; SD = 4.1, Female: Mean = 24.9; SD = 4.2, Mean = 24.2; SD = 4.5, respectively) (Table 1).

**Table 1:** General characteristics of the study population

Variables	K-MMSE^a^											
	Total		Male					Female				
	N	%	N	%	Mean	SD	***P-value***	N	%	Mean	SD	***P-value***
**Change of lifestyle (2006 → 2008)**							0.0001					<.0001
Good→Good	162	9.0	68	6.8	27.0	2.6		94	11.7	25.6	3.9	
Bad→Good	153	8.5	63	6.3	26.8	2.9		90	11.2	25.9	3.5	
Good→Bad	149	8.3	47	4.7	24.2	6.6		102	12.7	25.0	4.9	
Bad→Bad	1342	74.3	823	82.2	25.8	4.0		519	64.5	24.1	4.4	
**Age**							<.0001					<.0001
65–69	531	29.4	283	28.3	26.6	3.6		248	30.8	25.6	3.7	
70–74	709	39.3	398	39.8	26.3	3.5		311	38.6	24.6	4.5	
75–79	368	20.4	197	19.7	25.3	4.4		171	21.2	24.3	4.3	
80-	198	11.0	123	12.3	24.2	5.1		75	9.3	22.2	5.2	
**Economic activity**							0.0322					0.8536
Active	430	23.8	318	31.8	26.5	2.8		112	13.9	24.6	3.6	
Nonactive	1376	76.2	683	68.2	25.6	4.4		693	86.1	24.6	4.5	
**Region**							0.0003					0.0157
Urban area	830	46.0	435	43.5	26.3	3.9		395	49.1	24.9	4.2	
Rural area	976	54.0	566	56.5	25.6	4.1		410	50.9	24.2	4.5	
**Marital status**							0.1209					0.7045
Married	1334	73.9	906	90.5	26.0	4.0		428	53.2	24.8	4.1	
Single, widow, divorced, separated	472	26.1	95	9.5	25.0	4.1		377	46.8	24.3	4.7	
**Living arrangement**							0.4298					0.6053
Three generation family or more	227	12.6	94	9.4	25.4	4.0		133	16.5	23.9	5.0	
Two-generation family	384	21.3	215	21.5	26.1	4.3		169	21.0	24.8	4.5	
One-generation family	1195	66.2	692	69.1	25.9	3.9		503	62.5	24.7	4.2	
**Educational level**							<.0001					<.0001
Middle school or above	750	41.5	556	55.5	26.7	3.6		194	24.1	26.1	3.9	
Elementary school or below	1056	58.5	445	44.5	24.9	4.2		611	75.9	24.1	4.4	
**Household income**							0.2837					0.1881
Quartile 4 (high)	450	24.9	249	24.9	26.4	3.6		201	25.0	24.6	5.1	
Quartile 3	443	24.5	263	26.3	26.2	4.2		180	22.4	25.2	3.8	
Quartile 2	460	25.5	271	27.1	25.8	3.9		189	23.5	24.5	4.3	
Quartile 1 (low)	453	25.1	218	21.8	25.1	4.2		235	29.2	24.1	4.2	
**Participation in social activities**							<.0001					<.0001
2 or more	345	19.1	206	20.6	26.8	3.0		139	17.3	25.2	3.8	
1	979	54.2	531	53.0	26.2	3.6		448	55.7	25.2	4.1	
0	482	26.7	264	26.4	24.6	5.0		218	27.1	23.0	4.9	
**Chronic disease**							0.0168					0.3353
3 or more	233	12.9	111	11.1	25.0	5.2		122	15.2	23.9	5.5	
2	404	22.4	208	20.8	25.4	4.7		196	24.3	24.7	4.3	
1	636	35.2	342	34.2	26.0	3.7		294	36.5	24.7	4.2	
0	533	29.5	340	34.0	26.3	3.3		193	24.0	24.7	4.0	
**Depression**							<.0001					<.0001
No	864	47.8	533	53.2	26.9	2.8		331	41.1	25.6	3.6	
Yes	942	52.2	468	46.8	24.8	4.8		474	58.9	23.9	4.8	
**Disability status**							<.0001					0.0001
Disabled	251	13.9	180	18.0	24.1	6.0		71	8.8	22.2	6.2	
Nondisabled	1555	86.1	821	82.0	26.3	3.3		734	91.2	24.8	4.1	
**Lagged dependent variable**												
K-MMSE score of previous survey	1806	100.0	1001	100.0	27.5	1.9	<.0001	805	100.0	26.8	1.9	<.0001
**Total**	**1806**	**100.0**	**1001**	**100.0**	**25.9**	**4.0**		**805**	**100.0**	**24.6**	**4.4**	

^a^Cognitive function was measured using the K-MMSE

*K-MMSE* Korean Mini-Mental State Examination (score of 0–30), *SD* Standard deviation

Table 2 shows the relationship between lifestyle changes and cognitive functions that were repeatedly measured in two-year units from 2006 to 2016 as beta coefficients. All analyses were performed while adjusting for covariates. The reference group was the Bad→Bad group. Both males and females showed higher beta coefficients for the other groups than for the reference group. Among males, the Bad→Good and Good→Bad groups had higher beta coefficients compared to the reference group, albeit not significantly (***β*** = 0.274, SD = 3.522, *p* = 0.2700; ***β*** = 0.043, SD = 4.524, *p* = 0.8898, respectively). The Good→Good group, however, had a significantly higher beta coefficient than the reference group (***β*** = 0.509; SD = 3.245; p = 0.0077). Among females, all of the groups had significantly higher beta coefficients than the reference: Good→Good (***β*** = 0.813, SD = 4.654, p = 0.0005), Bad→Good (***β*** = 0.914; SD = 3.744; p < .0001), and Good→Bad (***β*** = 0.475, SD = 4.542, p = 0.0481). The Bad→Good group had the highest beta coefficient. Post-hoc analysis showed that, the Good→Good group of males and the Good→Bad group of females were not significant (Table 2).

**Table 2:** Generalized linear model using GEE with cognitive function in 2006–2016

Variables^b^	K-MMSE^a^					
	Male			Female		
	***β***	SD	***P-value***	***β***	SD	***P-value***
**Change of lifestyle**						
Good→Good	0.509	3.245	0.0077	0.813	4.654	0.0005*
Bad→Good	0.274	3.522	0.2700	0.914	3.744	<.0001*
Good→Bad	0.043	4.524	0.8898	0.475	4.542	0.0481
Bad→Bad	ref.			ref.		
**Age**						
65–69	0.933	5.236	0.0005*	1.652	6.579	<.0001*
70–74	0.610	7.726	0.0043	1.419	9.034	<.0001*
75–79	0.469	6.446	0.0079	0.865	7.249	<.0001*
80-	ref.			ref.		
**Economic activity**						
Active	0.675	4.487	<.0001	0.558	4.618	0.0138
Nonactive	ref.			ref.		
**Region**						
Urban area	0.223	6.453	0.1539	0.347	7.716	0.0696
Rural area	ref.			ref.		
**Marital status**						
Married	0.295	13.257	0.1803	−0.023	7.737	0.9059
Single, widow, divorced, separated	ref.			ref.		
**Living arrangement**						
Three generation family or more	−0.654	5.702	0.0292	−0.778	7.224	0.0150
Two-generation family	0.070	5.429	0.7221	−0.297	6.242	0.2114
One-generation family	ref.			ref.		
**Educational level**						
Middle school or above	0.787	7.838	<.0001	0.859	6.688	0.0003
Elementary school or below	ref.			ref.		
**Household income**						
Quartile 4 (high)	0.341	6.706	0.1086	0.680	7.459	0.0075
Quartile 3	0.211	6.497	0.2916	0.325	6.000	0.1490
Quartile 2	0.275	6.529	0.1642	0.330	5.591	0.1024
Quartile 1 (low)	ref.			ref.		
**Participation in social activities**						
2 or more	1.216	4.917	<.0001*	1.252	5.152	<.0001*
1	1.026	7.633	<.0001*	1.140	7.983	<.0001*
0	ref.			ref.		
**Chronic disease**						
3 or more	−0.531	6.635	0.0267	−0.170	8.136	0.5795
2	−0.140	5.869	0.4521	0.341	7.740	0.1700
1	−0.022	5.650	0.8936	0.431	7.891	0.0712
0	ref.			ref.		
**Depression**						
No	ref.			ref.		
Yes	−1.033	5.503	<.0001	−0.842	6.887	<.0001
**Disability status**						
Disabled	−2.126	6.668	<.0001	−2.488	6.953	<.0001
Nondisabled	ref.			ref.		
**Lagged dependent variable**						
MMSE score of previous survey	0.376	0.788	<.0001	0.336	0.701	<.0001
**Year**						
2008	−0.015	7.331	0.9475	−0.463	8.137	0.1068
2010	0.143	6.674	0.5246	−0.116	7.190	0.6693
2012	0.365	6.073	0.0883	0.366	5.967	0.1135
2014	−0.122	5.807	0.5737	−0.539	6.006	0.0242
2016	ref.			ref.		

*Significant *P*-value after Bonferroni correction

^a^Cognitive function was measured using the K-MMSE

^b^Age, economic status, region, marital status, living arrangement, educational level, household income, participation in social activities, chronic disease, depressive symptoms, disability status, and survey year were adjusted

*GEE* Generalized estimating equation, *K-MMSE* Korean Mini-Mental State Examination (score of 0–30), *SD* Standard deviation

**Fig. 2:**
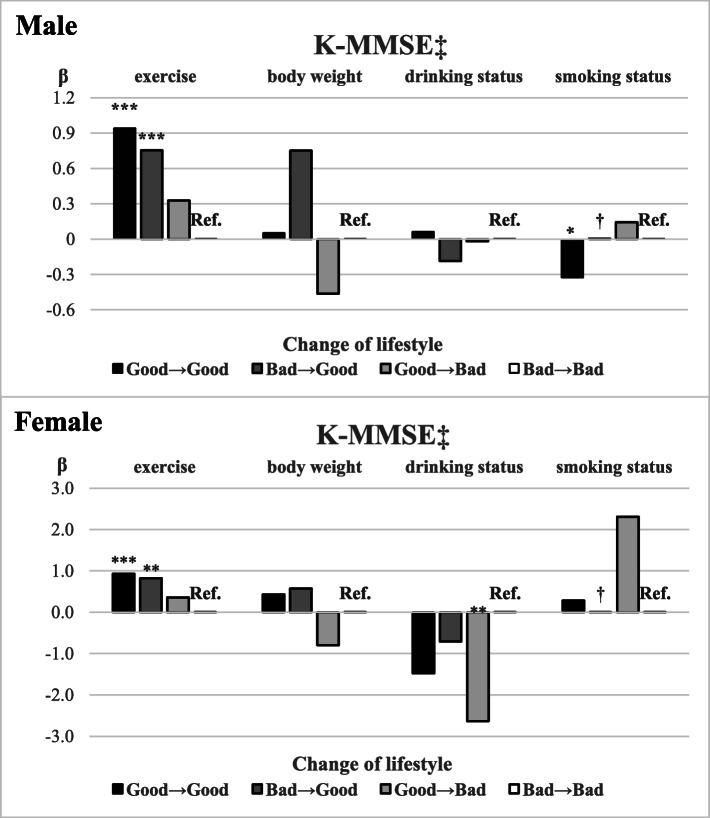
Results of subgroup analysis of the four lifestyle factors by sex**.** The results show the association between lifestyle change and cognitive function over two years. Statistically significant, *p < .05 ** < .01 *** < .0001. †The frequency is zero and cannot be calculated. ‡Cognitive function was measured using the K-MMSE. K-MMSE = Korean Mini-Mental State Examination (score of 0–30). The following variables were included as covariates in every lifestyle analysis: age, economic activity status, region, marital status, living arrangement, educational level, household income, participation in social activities, chronic disease, depression, disability status, survey year; and the four lifestyles were added as covariates to each other’s analysis (exercise: body weight, drinking status, and smoking status was added as covariates; body weight: exercise, drinking status, and smoking status was added as covariates; drinking status: exercise, body weight, smoking status; smoking status: exercise, body weight, drinking status was added as covariates)

Figure 2 shows the results of the subgroup analysis of the four lifestyle factors (i.e., exercise, body weight, alcohol drinking status, and smoking status). In both males and females, exercise was significantly associated with cognitive function: the Good→Good group had the highest beta coefficient compared to the reference group (Bad→Bad) (***β*** = 0.941; SD = 4.786; p < .0001, ***β*** = 0.930; SD = 4.638; p < .0001, respectively), followed by the Bad→Good group (***β*** = 0.753; SD = 4.190; p < .0001, ***β*** = 0.824; SD = 3.931; *p* = 0.0001, respectively) and Good→Bad group (***β*** = 0.328; SD = 4.910; *p* = 0.1145, ***β*** = 0.360; SD = 4.663; *p* = 0.1282, respectively). No significant result was found for body weight. In females, for alcohol drinking status, the Good→Bad group had a significantly lower beta coefficient than did the reference group (***β*** = − 2.636; SD = 2.915; p = 0.0011). For smoking status, there were no significant results for females. However, for males, the Good→ Good group had a significantly lower beta coefficient than did the reference group (***β*** = − 0.325; SD = 6.292; *p* = 0.0385). We could not obtain the results for the Bad→Good group in either sex, as no participant in this group had changes in smoking status (Fig. 2). The post-hoc analysis showed that all results were of the same significance, except for the significance of smoking status changes in males. According to changes in smoking status, the Good→Good group of males was not statistically significant according to the Bonferroni correction (Supplementary Table 1).

Table 3 shows the results of subgroups analysis for marital status, participation in social activities, and depression. The subgroup analysis by marital status showed that in both males and females, there was a tendency for a higher beta coefficient among single, widow, divorced, or separated participants compared to married participants. A subgroup analysis of participation in social activities for females showed that they had the highest beta coefficients from Bad→Good groups in all classes of this variable. In particular, females who did not participate in any social activities tended to have higher beta coefficients compared to females who participated (Good→Good: ***β*** = 1.614; SD = 4.270; p = 0.0017, Bad→Good: ***β*** = 1.817; SD = 3.945; *p* < .0001, Good→Bad: ***β*** = 0.536; SD = 4.592; *p* = 0.2817). Finally, regarding the subgroup analysis of depression, males without depression tended to have a higher beta coefficient compared to males who had depression. On the other hand, females who had depression tended to have a higher beta coefficient compared to females without depression (Table 3).

**Table 3:** Subgroup analysis using GEE of cognitive function^a^ with lifestyle change in 2006–2016

Variables^b^	Change of lifestyle									
	Good→Good			Bad→Good			Good→Bad			Bad→Bad
	***β***	SD	***P-value***	***β***	SD	***P-value***	***β***	SD	***P-value***	***β***
**Male**										
**Marital status**										
Married	0.409	3.214	0.0385	0.245	3.566	0.3559	−0.071	3.615	0.8337	Ref.
Single, widow, divorced, separated	1.627	3.394	0.0189	0.298	3.195	0.6687	0.652	3.416	0.3865	Ref.
**Participation in social activities**										
2 or more	0.342	2.699	0.2755	−0.100	2.702	0.7838	−0.580	2.246	0.3124	Ref.
1	0.467	2.785	0.0361	0.498	3.330	0.1274	0.660	3.455	0.0777	Ref.
0	0.668	4.202	0.2259	0.321	4.313	0.6295	−0.566	5.112	0.4104	Ref.
**Depression**										
No	0.554	2.983	0.0132	0.458	3.515	0.1372	0.607	3.277	0.0437	Ref.
Yes	0.209	3.582	0.5412	−0.042	3.002	0.9053	−0.683	3.491	0.2255	Ref.
**Female**										
**Marital status**										
Married	0.495	4.533	0.1394	0.863	3.981	0.0058	0.289	4.277	0.3602	Ref.
Single, widow, divorced, separated	1.002	4.704	0.0017*	0.888	3.491	0.0020*	0.557	3.754	0.1292	Ref.
**Participation in social activities**										
2 or more	0.566	4.545	0.2042	0.593	4.068	0.2672	−0.173	3.623	0.7488	Ref.
1	0.440	4.194	0.1151	0.500	3.543	0.0618	0.405	3.862	0.1906	Ref.
0	1.614	4.270	0.0017*	1.817	3.945	<.0001*	0.536	4.592	0.2817	Ref.
**Depression**										
No	0.573	4.159	0.0332	0.765	4.119	0.0199	−0.184	4.079	0.5697	Ref.
Yes	0.751	4.580	0.0380	0.864	3.621	0.0033*	0.716	4.185	0.0351	Ref.

*Significant *P*-value after Bonferroni correction

^a^Cognitive function was measured using the K-MMSE

^b^Age, economic status, region, marital status, living arrangement, educational level, household income, participation in social activities, chronic disease, depressive symptoms, disability status, and survey year were adjusted

GEE = generalized estimating equations, K-MMSE = Korean Mini-Mental State Examination (score of 0–30), SD = standard deviation

## Discussion

We examined the association between lifestyle changes and cognitive functions in South Korean elderly adults. When looking at cognitive function according to the changes in lifestyle over two years, as described in literature, we also found that participants who maintained a healthy lifestyle had significantly higher cognitive function compared to those with continually unhealthy lifestyle. Females showed a stronger association between lifestyle changes and cognitive functions compared to males. Especially, the females whose lifestyle improved (Bad→Good) for two years had the highest cognitive function compared to those with a continually unhealthy lifestyle over the same period, and the worsened (Good→Bad) group tended to have higher cognitive function compared to those with continually unhealthy lifestyle.

Although cognitive deterioration and dementia are not the same terms, deterioration of cognitive function is an essential factor in the diagnosis of dementia [28], and one of the most common measurement tools for assessing cognitive function is the MMSE [20, 21]. The dependency variables of our study were the K-MMSE scores, and they were calculated as a beta coefficient through analysis. Therefore, if the beta was positive, the MMSE score was high; and if it was negative, it could be interpreted as low. In other words, if the beta was negative, the cognitive function could be relatively low, and this may also be considered relatively more likely to cause dementia.

Previous studies have also found a relationship between multiple health-behavior factors and cognitive functions [14–16]. One cross-sectional study examined smoking, body weight, and exercise among US individuals aged 60 years or older, and found that cognitive function was the highest among those with all of the following factors: non-smoking, normal body weight, and sufficient exercise [14]. In particular, cognitive function was statistically significantly higher in those who engaged in sufficient amount of exercise. Other studies, including a nested case-control study of Japanese-American men [15] and a retrospective cohort study of European aged 60 years or over [16], investigated the association of lifestyle and genetic risk factors (ε4 allele of the apolipoprotein E gene) with the incidence of dementia. The former included smoking, diet, BMI and exercise as lifestyle factors, and the latter included smoking, exercise, diet, and drinking as lifestyle factors. These studies found that healthy lifestyle can reduce the risk of dementia. Our study differentiates itself from these studies in that it confirmed the association between changes in each lifestyle factor and cognitive function.

Our subgroup analysis revealed that exercise had the strongest association with cognitive function. To improve health, the WHO recommends that people aged 65 years and older engage in physical activities for more than 150 min a week [26]. In this study, exercise was included as one of the lifestyle factors, and the criteria for healthy exercise were set at more than 150 min per week, according to the WHO recommendation. Our findings support this recommendation and add evidence that proper amounts of regular exercise can help improve the cognitive functions of the elderly. In addition, our results support those of previous studies which showed that individuals engaging in little exercise or are inactive [29, 30] are particularly at risk of deteriorating cognitive function, and reaffirms the importance of steady exercise among elderly adults [19, 31, 32]. Findings from our study can be explained by mechanisms of the effect of exercise on the brain. During exercise, brain blood flow increases [33], and changes in neurotransmitters and neurotrophins occur [34]. In addition, new neurons in the hippocampus, a part of the brain that is related to learning and memory formation, are also increased [34].

Previous studies have shown that moderate amounts of alcohol can help cognitive function [35, 36]. Our study set one of the unhealthy lifestyles to drinking more than an appropriate amount of alcohol, and analysis results showed that cognitive functions were only low in women, and not in men who started drinking more than the appropriate amount of alcohol. These results were in line with those of previous studies [37, 38].

We also confirmed another modifiable, individual-level protective factor for cognitive function by sex: participation in social activities. This association was stronger for females than for males. Past studies have explained the relationship between social activities and cognitive function as follows. Social activity can increase the brain volume and improve global cognition; and is associated with memory, executive functioning, processing speed, and visuospatial perception [39]. Some previous studies also have shown that participation in social activities can prevent the decline in cognitive functions [40, 41], as demonstrated in our study.

Improving health is important for helping elderly adults extend their healthy lives. Our study may be meaningful in terms of public health, as it situates the power to motivate healthy behavior and proposes a way to prevent deterioration of cognitive function in the elderly. If the findings of this study can be used to mediate the cognitive function of the Korean elderly and achieve positive results to relieve the burden of an aged society, it can serve as a good example for other countries around the world, many of which are also becoming an aging society. It is necessary to confirm the causal relationship and effect of the intervention on cognitive function through further studies. Another strength of this study is that it used a nationwide data representing the elderly population in South Korea (i.e., the KLoSA), and we analyzed the data by sex. Furthermore, the data were longitudinal, which allowed us to analyze the impact of time on the other variables.

This study also had some limitations. First, although various efforts were made by the Korea Employment Information Service, a panel survey organization, to minimize bias (e.g., by educating survey investigators), they could not rule out response bias or recall bias, which might have influenced our results. In addition, the K-MMSE surveyed in this data is limited in that it does not reflect the educational level and age of the survey participants [20, 21]. Therefore, we tried to take this into consideration by adjusting the educational level and age of study subjects as covariates. Second, while we confirmed the association between the variables, we did not confirm whether the relationship was causal. Since this study was an observational study, its results cannot be interpreted as a causal relationship. Nevertheless, we do offer more accurate associations compared to cross-sectional studies, due to our use of panel data. Third, we likely omitted other relevant lifestyle factors for cognitive function. Still, we believe that our attempt to explore which lifestyle factor had the strongest association offered meaningful data. Finally, the subgroup analysis of smoking status should be carefully interpreted. Among males, while the results in the main analysis of the Good→Good group were significant and low, post-hoc analysis results were not statistically significant. Additionally, in both males and females, there were no participants who were classified as Bad→Good; therefore, our results for that group could not be calculated. Moreover, since South Korean women are more likely to show a response bias in their answers to smoking status questions [42], it is necessary to be careful in interpreting the results.

## Conclusions

We can conclude that continuing to follow a healthy lifestyle or improving one’s unhealthy lifestyle could help prevent or slow down cognitive decline. Regularly engaging in a proper amount of exercise can help the cognitive function of the elderly. In particular, females can experience positive effects on their cognitive function if they participate in social activities while maintaining healthy lifestyles, such as not drinking too much alcohol.

## Supplementary information

**Additional file 1 Table S1.** Generalized linear model through GEE analysis with cognitive function by lifestyle factors in 2008–2016.

## Data Availability

Data used in this study are available at: https://survey.keis.or.kr/eng/klosa/databoard/List.jsp

## Abbreviations

KLoSA: Korean Longitudinal Study of Aging
WHO: World Health Organization
ADLs: Activities of daily living
IADLs: Instrumental ADL
CAPI: Computer-assisted personal interviewing
K-MMSE: Korean Mini-Mental State Examination
BMI: Body mass index
CES-D10: 10-item Center for the Epidemiological Studies of Depression Short Form
ANOVA: Analysis of variance
GEE: Generalized estimating equation
SE: Standard errors
VIFs: Variance inflation factor
