# Supplementary material for: Association between change in lifestyle and cognitive functions among elderly Koreans: findings from the Korean longitudinal study of aging (2006–2016)
Source: BMC Geriatr. 2020 Aug 31;20:317. doi: 10.1186/s12877-020-01693-7 (PMC7457530; doi:10.1186/s12877-020-01693-7)
Supplement: Supplementary file 1 — Additional file 1 Table S1. Generalized linear model through GEE analysis with cognitive function by lifestyle factors in 2008–2016. [file 12877_2020_1693_MOESM1_ESM.docx]

| **sTable 1. Generalized linear model through GEE analysis with cognitive function by lifestyle factors in 2008-2016** | | | | | | | | | | |
| --- | --- | --- | --- | --- | --- | --- | --- | --- | --- | --- |
| **Variables‡** | **K-MMSE†** | | | | | | | | | |
|  | **Male** | | | | | **Female** | | | | |
|  | ***β*** | **SE** | **SD** | ***P*-value** | **Bonferroni*** | ***β*** | **SE** | **SD** | ***P*-value** | **Bonferroni*** |
| **Change of smoking status** |  |  |  |  |  |  |  |  |  |  |
| Good→Good | -0.325 | 0.157 | 6.292 | 0.0385 | 0.1154 | 0.284 | 0.433 | 24.643 | 0.5128 | 1.0000 |
| Bad→Good | - | - | - | - |  | - | - | - | - |  |
| Good→Bad | 0.144 | 0.568 | 3.938 | 0.8001 | 1.0000 | 2.311 | 1.232 | 3.483 | 0.0606 | 0.1817 |
| Bad→Bad | Ref. |  |  |  |  | Ref. |  |  |  |  |
| **Change of alcohol status** |  |  |  |  |  |  |  |  |  |  |
| Good→Good | 0.062 | 0.179 | 9.798 | 0.7309 | 1.0000 | -1.474 | 0.775 | 44.740 | 0.0571 | 0.3425 |
| Bad→Good | -0.186 | 0.255 | 5.072 | 0.4653 | 1.0000 | -0.707 | 1.001 | 4.129 | 0.4802 | 1.0000 |
| Good→Bad | -0.019 | 0.266 | 4.003 | 0.9445 | 1.0000 | -2.636 | 0.809 | 2.915 | 0.0011 | 0.0067* |
| Bad→Bad | Ref. |  |  |  |  | Ref. |  | 0.000 |  |  |
| **Change of BMI** |  |  |  |  |  |  |  |  |  |  |
| Good→Good | 0.052 | 0.394 | 23.675 | 0.8953 | 1.0000 | 0.428 | 0.408 | 22.432 | 0.2947 | 1.0000 |
| Bad→Good | 0.751 | 0.457 | 4.410 | 0.1003 | 0.6021 | 0.577 | 0.527 | 5.527 | 0.2736 | 1.0000 |
| Good→Bad | -0.462 | 0.547 | 6.581 | 0.3983 | 1.0000 | -0.794 | 0.619 | 6.432 | 0.1994 | 1.0000 |
| Bad→Bad | Ref. |  |  |  |  | Ref. |  |  |  |  |
| **Change of physical activity** |  |  |  |  |  |  |  |  |  |  |
| Good→Good | 0.941 | 0.155 | 4.786 | <.0001 | <.0001* | 0.930 | 0.219 | 4.638 | <.0001 | 0.0001* |
| Bad→Good | 0.753 | 0.186 | 4.190 | <.0001 | 0.0003* | 0.824 | 0.215 | 3.931 | 0.0001 | 0.0008* |
| Good→Bad | 0.328 | 0.208 | 4.910 | 0.1145 | 0.6873 | 0.360 | 0.236 | 4.663 | 0.1282 | 0.7691 |
| Bad→Bad | Ref. |  |  |  |  | Ref. |  |  |  |  |
| *Significant P‐value after Bonferroni correction | | | | | | | | | | |
| †Cognitive functions were measured by scores on the K-MMSE. | | | | | | | | | | |
| ‡Adjusted by age, economic status, region, marital status, living arrangement, educational level, household income, participation in social activities, chronic disease, depressive symptoms, disability status, and survey year variables. | | | | | | | | | | |
| GEE= generalized estimating equations, K-MMSE = Korean version of the Mini-Mental State Examination (0 - 30 scores), SE = standard error, SD = standard deviation | | | | | | | | | | |
